# Fruit and vegetable consumption and proinflammatory gene expression from peripheral blood mononuclear cells in young adults: a translational study

**DOI:** 10.1186/1743-7075-7-42

**Published:** 2010-05-13

**Authors:** Helen Hermana M Hermsdorff, María Ángeles Zulet, Blanca Puchau, José Alfredo Martínez

**Affiliations:** 1 Department of Nutrition, Food Science, Physiology and Toxicology, University of Navarra, Pamplona, Spain

## Abstract

**Background:**

Fruits and vegetables are important sources of fiber and nutrients with a recognized antioxidant capacity, which could have beneficial effects on the proinflammatory status as well as some metabolic syndrome and cardiovascular disease features. The current study assessed the potential relationships of fruit and vegetable consumption with the plasma concentrations and mRNA expression values of some proinflammatory markers in young adults.

**Methods:**

One-hundred and twenty healthy subjects (50 men/70 women; 20.8 ± 2.6 y; 22.3 ± 2.8 kg/m^2^) were enrolled. Experimental determinations included anthropometry, blood pressure and lifestyle features as well as blood biochemical and inflammatory measurements. The mRNA was isolated from peripheral blood mononuclear cells (PBMC) and the gene expression concerning selected inflammatory markers was assessed by quantitative real-time PCR. Nutritional intakes were estimated by a validated semi-quantitative food-frequency questionnaire.

**Results:**

The highest tertile of energy-adjusted fruit and vegetable consumption (>660 g/d) was associated with lower plasma concentrations of C-reactive protein (CRP) and homocysteine and with lower *ICAM1, IL1R1, IL6, TNFα *and *NFκB1 *gene expression in PBMC (*P *for trend < 0.05), independently of gender, age, energy intake, physical activity, smoking, body mass index, systolic blood pressure and circulating non-esterified fatty acids. In addition, plasma CRP, homocysteine and TNFα concentrations and *ICAM1, TNFα *and *NFκB1 *gene expression in PBMC showed a descending trend as increased fiber intake (>19.5 g/d) from fruits and vegetables (*P *for trend < 0.05). Furthermore, the participants within the higher tertile (>11.8 mmol/d) of dietary total antioxidant capacity showed lower plasma CRP and mRNA values of *ICAM1, IL1R1, IL6, TNFα *and *NFκB1 *genes (*P *for trend < 0.05). The inverse association between fruit and vegetable consumption and study proinflammatory markers followed the same trend and remained statistically significant, after the inclusion of other foods/nutrients in the linear regression models.

**Conclusion:**

A higher fruit and vegetable consumption was independently associated not only with reduced CRP and homocysteine concentrations but also with a lower mRNA expression in PBMC of some relevant proinflammatory markers in healthy young adults.

## Background

A low-chronic inflammatory status is a recognized link between excessive adiposity and metabolic syndrome features, diabetes, and atherosclerosis [1,2]. In fact, a number of studies has demonstrated an increased expression of transcription nuclear factors such as nuclear-factor-kappa-B (NFκB) as well as of interleukins (IL) and tumor necrosis factor-alpha (TNFα) in obese subjects [1,3]. These changes appear to involve a higher production of proinflammatory and pro-atherogenic molecules such as C-reactive protein (CRP), homocysteine, selectins and adhesion molecules as well as some cytokines [1,3]. Furthermore, circulating peripheral blood mononuclear cells (PBMC) may have an important role in all these complex inflammatory processes, which are mediated by transcriptional nuclear-factors, cytokines and other pro-atherogenic molecules [4].

In turn, fruits and vegetables contain components such as plant proteins, potassium, magnesium, fiber, and others compounds with antioxidant capacity, whose consumption may reduce the risk of suffering metabolic syndrome manifestations [5-8]. Thus, the intake of fruits and vegetables has been related to marked reductions in proinflammatory and oxidative stress markers [9-12]. These previous findings indicate that a targeted emphasis on fruit and vegetable consumption could potentially help individuals in preventing and/or reducing the onset of cardiovascular diseases and metabolic syndrome complications by means of a beneficial modulation of low-grade inflammation and oxidative stress mediated processes.

In this context, nutrigenomic studies have demonstrated the healthy effect of specific nutrients and calorie-restriction on PBMC proinflammatory gene expression [13,14]. However, the effect of fruit and vegetable consumption on the expression of proinflammatory-related molecules in PBMC has not yet been apparently investigated.

Overall, the present study assessed the potential association of fruit and vegetable consumption with plasma concentrations of CRP, homocysteine, IL6, and TNFα as well as gene expression profiles, which were assessed through messenger RNA (mRNA) levels of genes encoding intercellular adhesion molecule-1 (*ICAM1*), interleukin-1 receptor-type 1 (*IL1R1*), interleukin-6 (*IL6*), tumor necrosis factor-alpha (*TNFα*), subunit-1 or p50 (*NFκB1*) and subunit-3 or p65 (*RELA*) of NFκB in PBMC, from young adults.

## Methods

### Subjects

One hundred twenty (50 men/70 women) subjects with a mean age of 20.8 ± 2.6 (range 18-30) years old and mean body mass index of 22.3 ± 2.8 (range 18.5-30.5) kg/m^2 ^participated in this study. The volunteers were recruited through magazines, radio, web page and intranet tools from the University of Navarra. Exclusion criteria included previous inflammatory, heart, and respiratory diseases, hormonal treatment or drug use that affect glucose metabolism, alcohol and drug dependence, history of diets for weight loss or unstable weight in the last three months as examined by a trained physician. Each participant signed a written informed consent, which was previously approved by the Investigation Ethics Committee of the Clínica Universidad de Navarra (79/2005), in accordance with the principles of the Helsinki Declaration.

### Dietary intake assessment

Assessment and information about dietary intake were obtained by the questionnaire of the Seguimiento Universidad de Navarra (SUN) Study [15]. This semi-quantitative food frequency questionnaire, which is validated for Spanish people [16], includes 136 items, considering 13 fruit items (oranges, banana, apple, pear, strawberry, grapefruit, peach/apricot/nectarine, cherry/plum, watermelon, melon, mango, papaya, and kiwi) and 11 vegetable items (spinach, lettuce, cauliflower/broccoli, tomatoes, carrots, green beans, peppers, cabbage, aubergines, asparagus, and 'gazpacho') related to fruit and vegetable consumptions, respectively. Also, natural orange juice and other natural fruit juices were also separately accounted in the questionnaire.

Nutrient intake was estimated using an *ad hoc *computer program specifically developed for this aim [15]. A dietitian updated the nutrient data bank using the latest available information included in the food composition tables for Spain [17,18]. Nutrient intake was calculated as frequency × nutrient composition of each portion size for each consumed food item, where frequencies were measured in nine frequency categories (6+/d, 4 to 6/d, 2 to 3/d, 1/d, 5 to 6/wk, 2 to 4/wk, 1/wk, 1 to 3/mo, never or almost never) for each food item. This questionnaire has been successfully applied to estimate dietary intake and specific nutrients in relation to proinflammatory outcomes [19-21].

Furthermore dietary total antioxidant capacity (TAC), expressed as mmol/d, was calculated by a proxy previously validated to this food-frequency questionnaire [20].

### Clinical and biochemical assessments

Anthropometrical measurements were performed according to previously described procedures [19]. Thus, BMI was calculated by the quotient between weight (kg) and the squared height (m^2^), which was applied to categorize normal-weight (18.5-24.9 kg/m^2^), overweight (25-29.9 kg/m^2^), and obesity (BMI ≥ 30 kg/m^2^) subjects, according to the Spanish Society for Obesity Study criteria [22]. Blood systolic and diastolic pressures were measured according to WHO criteria [23].

Venous blood samples were drawn after a 12 h overnight fast by venipuncture. The EDTA-plasma and serum samples were separated from whole blood by centrifugation at 2,205 *g*, 15 min, at 5°C (Model 5804R, Eppendorf, Hamburg, Germany), and were frozen immediately at -80°C until assay. Plasma concentrations of triglycerides, total cholesterol, HDL-c, glucose (Horiba ABX Diagnostics, Montpellier, France), non-esterified fatty acid (NEFA) (Wako Chemicals GmbH, Neuss, Germany), and homocysteine (Demitec Diagnostic GmbH, Kiel-Wellsee, Germany) were measured by specific colorimetric assays, using an automated analyzer system (COBAS MIRA, Roche, Basel, Switzerland). Serum fasting insulin was measured by an ELISA kit (Mercodia, Uppsala, Sweden). Insulin resistance was estimated by the HOMA-IR, which was calculated as follows: HOMA-IR = [fasting glucose (mmol/l) × fasting insulin (μIU/l)]/22.5 [24]. Plasma concentrations of CRP, high-sensitive IL6 (hs-IL6), and TNFα (hs-TNFα) were measured by ELISA kits using an automated analyzer system (Triturus, Grifols, Barcelona, Spain). CRP was measured by using an Immundiagnostik AG kits, (Bensheim, Germany) and the cytokines by Quantikine immunoassay kits (hs-IL6 and hs-TNFα) from R&D Systems (Minneapolis, USA). In our laboratory, all the inter- and intra-assay variabilities were <10% selected for determinations.

PBMC were isolated by differential centrifugation (450 *g*, 30 min, at 20°C) by using PMN medium (Robbins Scientific Corporation, Sunnyvale, CA, USA), and total RNA from PBMC was extracted and subsequently purified by DNase treatment (DNA-free kit, Ambion/Applied Biosystems, Austin, TX, USA) as previously described [25]. Quantitative real-time PCR was performed by using an ABI PRISM 7000 HT Sequence Detection System as described by the manufacturer (Applied Biosystems, Foster City, CA, USA). Taqman probes for genes (*ICAM1, IL1R1, IL6, TNFα, NFκB1, RELA*) were also supplied by Applied Biosystems (Foster City, CA, USA). Gene expression levels were assessed and normalized by using 18s rRNA as internal control following previously described protocols [26,27]. The fold change (2^-ddCt^) in the target genes, normalized to 18S and relative to the lowest expression profile, was calculated for each sample according to the manufacturer's guidelines (Applied Biosystems, Foster City, CA, USA).

### Other variables assessment

Regarding to other covariables, the SUN questionnaire also was used for collecting the information about lifestyle details as smoking status (never, former, and current smokers) and physical activity practice (Yes/No and volume of activity). To quantify the volume of activity, an activity metabolic equivalent (MET) index was computed by assigning a multiple of resting metabolic rate (MET score) to each activity [28]. Metabolic equivalents were estimated as the ratio of energy expended during each specific activity to resting metabolic rate and they are independent of body weight. Time spent in each of the activities was multiplied by the MET score specific to each activity, and then interpreted over all activities obtaining a value of overall weekly MET/h [29].

### Statistical analysis

Results are shown as mean ± standard deviations (SD). The Shapiro-Wilk test was used to determine variable distribution. Comparisons between three groups were performed by ANOVA one-factor tests, while the post-hoc Tukey test was used concerning multiple comparisons. Dietary intakes were adjusted for the daily energy intake by the residuals method, applying separate models among women and men [30]. To assess the associations of fruit and vegetable consumption with plasma concentration and mRNA expression of proinflammatory markers, we categorized the participants by tertiles of food-group consumption. Means and SD were calculated for each related variable within each tertile of fruit and vegetable consumption. Linear trends were assessed by assigning the median value to each tertile of fruit and vegetable consumption, and modeling these values as a continuous variable. Data in the models were controlled for gender, age (years), BMI (kg/m^2^), daily energy intake (kcal/d), physical activity during leisure time (METs-hour per week), smoking (never, former, and current smokers), systolic blood pressure (mmHg), and non-esterified fatty acid concentration (mmol/l). The same procedure was performed in order to analyze the relationships of dietary TAC and dietary fiber (from fruit and vegetable intake) with proinflammatory markers. In addition, we categorized the participants by quintiles of fruit and vegetable consumption, and the ratios between the mean values of selected proinflammatory marker concentrations (CRP, homocysteine, IL6 and TNFα), computed as the quotient between fifth and first quintiles of this food-group consumption were calculated.

Furthermore, we used stepwise multiple regression [30] to identify the main variability food-items concerning fruit and vegetable consumption of participants of this study. Finally, statistical analyses were performed with SPSS 15.0 software (SPSS Inc., Chicago, IL, USA) for Windows XP (Microsoft, USA). A *P*-value < 0.05 was considered as statistically significant.

## Results

**Figure 1:**
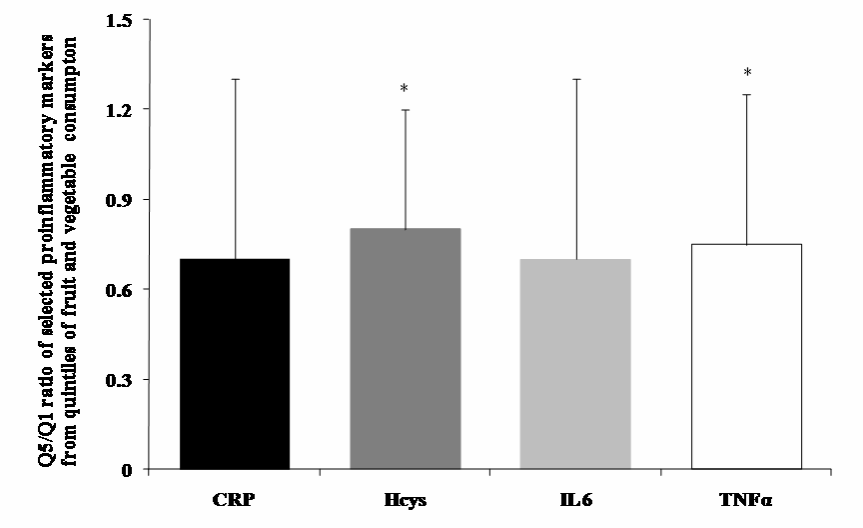
**Ratios of protein levels of selected proinflammatory markers, calculated from the fifth quintile (Q5: > 1150 g/d; n = 24), as compared to the first quintile (Q1: < 380 g/d; n = 24) of energy-adjusted fruit and vegetable consumption**. *P < 0.05, Q1 vs. Q5, from post hoc Tukey test to correct for multiple comparisons, after performing ANOVA one-factor test (n = 120). CRP, C-reactive protein; Hcys, homocysteine; IL6, interleukin-6; TNFα, tumor necrosis factor-alpha

Anthropometrical, clinical, and metabolic characteristics were examined by tertiles of fruit and vegetable consumption (Table 1). Thus, subjects within the highest tertile showed significantly lower values of BMI, waist circumference, systolic and diastolic blood pressure, as compared with those of the lowest tertile as well as lower homocysteine concentrations (*P *< 0.05). The circulating levels of other proinflammatory markers followed a decreasing trend, when analyzed with such criteria (Table 1). In addition, the ratios of mean concentrations of CRP, homocysteine, TNFα, and IL6, calculated as the quotient between values included in the fifth and in the first quintile of fruit and vegetable consumption, followed the same pattern, being the values of the highest quintile about 60-70% of the levels concerning the lowest quintile (Figure 1), with statistical significant differences (*P *< 0.05) reached for homocysteine and TNFα concentrations.

**Table 1:** Anthropometrical, clinical and metabolic characteristics of participants, according to tertiles (T) of energy-adjusted fruit and vegetable consumption (n = 120)

Energy-adj. fruit and vegetable consumption	T1 (n = 39) < 384 g/d	T2 (n = 42) 384-660 g/d	T3 (n = 39) > 660 g/d	*P*-value^a^
Age (y)	21 ± 2^b^	21 ± 3	21 ± 3	0.509
Body mass index (kg/m^2^)	22.6 ± 3.0^c^	22.4 ± 2.8	21.7 ± 2.89	*0.028*
Waist circumference (cm)	76.8 ± 8.3^c^	74.3 ± 8.8	70.9 ± 8.3	*0.025*
Systolic BP (mmHg)	117 ± 11^c^	115 ± 11	111 ± 10	*0.015*
Diastolic BP (mmHg)	67 ± 7^c^	66 ± 7	63 ± 8	*0.018*
Glucose (mg/dl)	85.8 ± 7.6	85.4 ± 9.4	85.2 ± 7.3	0.155
Insulin (μIU/l)	7.2 ± 2.9	8.2 ± 3.9	7.4 ± 3.5	0.966
HOMA-IR	1.6 ± 0.7	1.8 ± 0.9	1.6 ± 0.7	0.662
Total cholesterol (mg/dl)	175 ± 26	179 ± 26	169 ± 29	0.294
LDL-c (mg/dl)	102 ± 21	104 ± 23	101 ± 27	0.824
HDL-c (mg/dl)	60 ± 15	61 ± 12	56 ± 12.3	0.242
Triglycerides (mg/dl)	67 ± 29	71 ± 27	63 ± 23	0.385
NEFA (mmol/l)	0.43 ± 0.22	0.37 ± 0.14	0.32 ± 0.22	0.116
CRP (mg/l)	1.3 ± 0.8	1.1 ± 0.8	1.0 ± 0.7	0.217
Homocysteine (μmol/l)	15.0 ± 7.6^c^	12.6 ± 3.2	10.0 ± 3.2	*0.013*
IL6 (pg/ml)	1.3 ± 0.5	1.2 ± 0.6	1.2 ± 0.8	0.607
TNFα (pg/ml)	2.3 ± 1.2	1.8 ± 1.5	1.6 ± 1.2	0.577
Self-reported PA n, (%)	21 (61.5)	24 (50)	25 (66)	0.237
MET (h-week)	35.7 ± 32.7	38.2 ± 27.3	42.7 ± 27.5	0.480
Smoking Never n, (%)	22 (56.4)	28 (66.7)	27 (71.1)	0.269
Former n, (%)	4 (10.3)	5 (11.9)	3 (7.9)	
Current n, (%)	13 (33.3)	9 (21.4)	8 (21.1)	

^a^*P*-value from ANOVA test or χ^2 ^test, for continuous or categorical variables, respectively.

^b^Continuous variables are presented as mean values ± standard deviation, while categorical variables are presented as absolute number and frequencies.

^c ^*P *< 0.05, T1 vs. T3; from post hoc Tukey test to correct for multiple comparisons.

BP, blood pressure; HOMA-IR, insulin resistance index; LDL-c, low density lipoprotein-cholesterol; HDL-c, high density lipoprotein-cholesterol; NEFA, non-esterified fatty acid; CRP, C-reactive protein; IL6, interleukin-6; TNFα, tumor necrosis factor-alpha; MET, activity metabolic equivalent.

Moreover, those participants who were included in the third tertile also presented higher intake values for vegetable protein, potassium, magnesium, and vitamin C, as compared with subjects in the lowest tertile of fruit and vegetable consumption. Interestingly, fiber and dietary TAC also were statistically higher in those individuals included in the highest tertile of fruit and vegetable consumption (Table 2). Moreover, the more consumed fruit and vegetable by the participants were oranges and tomatoes (*R*^2 ^= 0.45 and 0.42, respectively). Other important contributing food items to the dietary intake are listed in the Table 3, which explained 91 and 94% of total variability in fruit and vegetable intake, respectively.

**Table 2:** Food and nutrients consumption, according to tertiles (T) of energy-adjusted fruit and vegetable consumption (n = 120)

Energy-adj. fruit and vegetable consumption	T1 (n = 39) < 384 g/d	T2 (n = 42) 384-660 g/d	T3 (n = 39) > 660 g/d	*P*-value^a^
Energy intake (kcal)	2645 ± 1110	2573 ± 899	2749 ± 920	0.703
Carbohydrate (% EI)	41.8 ± 6.5	43.0 ± 5.2	44.1 ± 6.5	0.207
Protein (%EI)	16.4 ± 2.5^b^	17.2 ± 2.6	18.0 ± 2.4	*0.012*
Vegetable protein (g)	29.9 ± 15.4^b^	31.6 ± 12.2	38.2 ± 11.8	*0.001*
Lipid (%EI)	40.4 ± 5.9^b^	38.2 ± 4.5	37.0 ± 7.2	*0.025*
PUFA (%EI)	6.1 ± 2.0^b,c^	5.2 ± 1.4	5.2 ± 1.4	*0.017*
MUFA (%EI)	16.9 ± 3.3	16.2 ± 3.2	16.2 ± 4.5	0.576
SFA (%EI)	14.8 ± 3.4^b^	13.4 ± 1.9	12.5 ± 2.8	*0.001*
Alcohol (g/d)	5.5 ± 8.1	5.3 ± 5.5	3.8 ± 4.6	0.339
Fruit juices (ml/d)	48.4 ± 70.0	79.3 ± 125.5	99.6 ± 116.8	0.076
Cereals (g/d)	197.5 ± 116.7	180.9 ± 106.6	174.8 ± 71.6	0.537
Legumes (g/d)	18.5 ± 13.4	27.4 ± 27.2	23.6 ± 17.9	0.119
Nuts (g/d)	11.7 ± 28.2	15.7 ± 6.1	15.6 ± 29.1	0.135
Meats (g/d)	205.0 ± 95.5	201.4 ± 101.9	213.4 ± 128.1	0.865
Dairy products (g/d)	335.1 ± 303.0	221.3 ± 177.6	238.9 ± 225.5	0.056
Olive oil (g/d)	15.8 ± 13.9	18.9 ± 17.6	20.6 ± 18.1	0.385
Potassium (g/d)	3.8 ± 1.7^b^	4.6 ± 1.3^d^	6.1 ± 2.0	*<0.001*
Magnesium (mg/d)	369.7 ± 163.1^b^	406.4 ± 124.2^d^	504.7 ± 163.4	*<0.001*
Vitamin C (mg/d)	153.2 ± 83.3^b,c^	253.4 ± 108.0^d^	381.2 ± 150.1	*<0.001*
Dietary fiber (g/d)	17.6 ± 10.3^b,c^	24.7 ± 10.3^d^	34.12 ± 13.3	*<0.001*
Dietary TAC (mmol/d)	6.0 ± 3.6^b,c^	8.0 ± 2.8^d^	11.4 ± 4.8	*<0.001*

^a^*P*-value from ANOVA test.

^b^*P *< 0.05, T1 vs. T3; ^c^*P *< 0.05, T1 vs. T2; ^d^*P *< 0.05, T2 vs. T3, from post hoc Tukey test to correct for multiple comparisons.

PUFA, polyunsaturated fatty acid; MUFA, monounsaturated fatty acid; SFA, saturated fatty acid; TAC, total antioxidant capacity.

**Table 3:** Main fruit and vegetable items consumed by participants (n = 120)

Fruits	R^2^	Cumulative R^2^	Vegetables	R^2^	Cumulative R^2^
Orange	0.45	0.45	Tomato	0.42	0.42
Cherry	0.15	0.60	Lettuce	0.32	0.74
Apple	0.15	0.75	Aubergine	0.07	0.81
Peach	0.07	0.82	Gazpacho	0.03	0.84
Pear	0.03	0.85	Spinach	0.03	0.87
Kiwi	0.02	0.87	Asparagus	0.02	0.89
Melon	0.01	0.88	Green beans	0.02	0.91
Watermelon	0.01	0.89	Carrot	0.01	0.92
Banana	0.01	0.90	Green pepper	0.01	0.93
Strawberry	0.01	0.91	Cabbage	0.01	0.94

Regarding proinflammatory markers, individuals on the highest tertile of fruit and vegetable consumption had statistically lower (*P *for trend < 0.05) values to plasma concentration of CRP and homocysteine (Table 4), after adjusting for several covariates. Interestingly, the concentrations of CRP, homocysteine, and TNFα were significantly lower (*P *< 0.05) across tertiles of fiber intake from fruits and vegetables, while levels of CRP were also lower across tertiles of increasing dietary TAC values (Table 4).

**Table 4:** Proinflammatory plasma concentration with respect to tertiles (T)^a ^of energy-adjusted fruit and vegetable consumption, fiber from fruit and vegetable consumption and dietary TAC (n = 120)

Proinflammatory plasma concentration	Energy-adj. fruit and vegetable consumption			*P *for trend
		
	T1 (<384 g/d)	T2 (384-660 g/d)	T3 (>660 g/d)	
CRP (mg/l)	1.2 ± 0.8	1.1 ± 0.8	1.0 ± 0.7	*0.027*
Homocysteine (μmol/l)	15.0 ± 7.6	10.6 ± 3.2	10.0 ± 3.2	*0.007*
IL6 (pg/ml)	1.3 ± 0.5	1.2 ± 0.6	1.2 ± 0.8	0.480
TNFα (pg/ml)	2.3 ± 1.2	1.8 ± 1.5	1.6 ± 1.2	0.484

	**Energy-adj. fiber from fruits and vegetables**			***P *for trend**
		
	**T1** **(<6.0 g/d)**	**T2** **(6.0-19.5 g/d)**	**T3** **(>19.5 g/d)**	

CRP (mg/l)	1.3 ± 0.7	1.1 ± 0.9	0.9 ± 0.7	*0.024*
Homocysteine (μmol/l)	14.4 ± 6.9	10.2 ± 2.6	9.8 ± 2.9	*0.008*
IL-6 (pg/ml)	1.5 ± 0.8	1.3 ± 0.6	1.2 ± 0.5	0.963
TNFα (pg/ml)	2.3 ± 2.8	2.2 ± 1.9	1.9 ± 1.8	*0.017*

	**Energy-adj. dietary TAC**			***P *for trend**
		
	**T1** **(<5.3 mmol/d)**	**T2** **(5.3-11.8 mmol/d)**	**T3** **(>11.8 mmol/d)**	

CRP (mg/l)	1.3 ± 0.7	1.2 ± 0.9	1.1 ± 0.7	*0.045*
Homocysteine (μmol/l)	12.7 ± 5.9	11.4 ± 4.6	10.2 ± 3.3	0.088
IL-6 (pg/ml)	1.3 ± 1.0	1.4 ± 0.7	1.3 ± 0.7	0.393
TNFα (pg/ml)	2.1 ± 1.6	1.9 ± 1.8	1.7 ± 1.4	0.077

^a ^Tertile 1, n = 39; Tertile 2, n = 42; Tertile 3, n = 39.

*P *for trend from linear regression model, adjusting for gender, age, BMI, energy intake, physical activity, smoking, systolic blood pressure and non-esterified fatty acid concentration. TAC, total antioxidant capacity; CRP, C-reactive protein; IL6, interleukin-6; TNFα, tumor necrosis factor-alpha.

Interestingly, individuals on the highest tertile of fruit and vegetable consumption had statistically lower (*P *for trend < 0.05) values to mRNA expression of *ICAM1, IL1R1, IL6, TNFα*, and *NFκB1(p50) *in PBMC, independent from assumed confounding factors (Table 5). In addition, *ICAM1, IL1R1, IL6, TNFα*, and *NFκB1(p50) *gene expressions were significantly lower in the subjects included in the highest tertile of dietary TAC (Table 5), while mRNA levels of *ICAM1*, *TNFα*, and *NFκB1(p50) *genes were markedly reduced in the third tertile of dietary fiber from fruit and vegetable consumption (Table 5).

**Table 5:** mRNA levels of proinflammatory gene expression from PBMC with respect to tertiles (T)^a ^of energy-adjusted fruit and vegetable consumption, fiber from fruit and vegetable consumption and dietary TAC (n = 120)

Proinflammatory gene expression (arbitrary units)	Energy-adj. fruit and vegetable consumption			*P *for trend
		
	T1 (<384 g/d)	T2 (384-660 g/d)	T3 (>660 g/d)	
*ICAM1*	2.03 ± 0.43	1.81 ± 0.40	1.75 ± 0.50	*0.002*
*IL1R1*	2.10 ± 0.58	1.95 ± 0.53	1.80 ± 0.56	*0.003*
*IL6*	2.02 ± 0.52	1.94 ± 0.61	1.75 ± 0.79	*0.005*
*TNFα*	1.96 ± 0.53	1.81 ± 0.49	1.68 ± 0.65	*0.025*
*NFκB1(p50)*	6.28 ± 4.81	5.08 ± 3.30	3.60 ± 1.90	*0.004*
*RELA (p65)*	3.49 ± 2.70	2.48 ± 1.85	2.17 ± 2.10	0.055

	**Energy adj. fiber from fruits and vegetables**			***P *for trend**
		
	**T1** **(<6.0 g/d)**	**T2** **(6.0-19.5 g/d)**	**T3** **(>19.5 g/d)**	

*ICAM1*	1.94 ± 0.38	1.87 ± 0.47	1.77 ± 0.47	*0.007*
*IL1R1*	1.99 ± 0.54	1.95 ± 0.59	1.88 ± 0.57	0.263
*IL6*	2.0 ± 0.52	1.90 ± 0.74	1.81 ± 0.63	0.156
*TNFα*	1.89 ± 0.49	1.81 ± 0.64	1.74 ± 0.55	*0.012*
*NFκB1(p50)*	5.92 ± 2.36	5.30 ± 3.51	3.67 ± 2.53	*0.011*
*RELA (p65)*	3.05 ± 2.36	2.94 ± 2.31	2.17 ± 1.62	0.104

	**Energy-adjusted dietary TAC**			***P *for trend**
		
	**T1** **(<5.3 mmol/d)**	**T2** **(5.3-11.8 mmol/d)**	**T3** **(>11.8 mmol/d)**	

*ICAM1*	1.91 ± 0.42	1.87 ± 0.47	1.79 ± 0.48	*0.038*
*IL1R1*	1.99 ± 0.57	1.95 ± 0.54	1.88 ± 0.59	*0.020*
*IL6*	1.97 ± 0.67	1.92 ± 0.65	1.85 ± 0.63	*0.042*
*TNFα*	1.89 ± 0.60	1.81 ± 0.51	1.75 ± 0.59	*0.031*
*NFκB1(p50)*	5.65 ± 4.51	5.21 ± 3.83	4.05 ± 2.17	*0.035*
*RELA (p65)*	3.05 ± 2.53	2.85 ± 1.95	2.30 ± 1.85	0.088

^a ^Tertile 1, n = 39; Tertile 2, n = 42; Tertile 3, n = 39.

*P *for trend from linear regression model, adjusting for gender, age, BMI, energy intake, physical activity, smoking, systolic blood pressure and non-esterified fatty acid concentration. TAC, total antioxidant capacity; ICAM1, intercellular adhesion molecule-1; IL1R1, interleukin-1 receptor-1; IL6, interleukin-6; NFκB1, nuclear factor-nuclear-kappa-B (p50); PBMC, peripheral blood mononuclear cells; RELA, nuclear factor-nuclear-kappa-B (p65); TNFα, tumor necrosis factor-alpha.

Furthermore, other analyses were performed in order to evaluate the association of fruit and vegetable consumption and proinflammatory markers. Since fruit and vegetable consumption correlated with other foods/nutrients, the association between fruit and vegetable consumption (as independent variable) and proinflammatory markers (as outcomes) were analyzed, using linear regression models, additionally adjusted for vegetable protein, potassium, magnesium, total fat, polyunsaturated fatty acid or saturated fatty acid intake as well as for dietary TAC and fiber, as continuous variables. The inverse association between fruit and vegetable intake and proinflammatory markers remained statistically significant (data not shown). Moreover, we performed additional analyses including fruit juices in the categories of fruit and vegetable consumption, which did not change the interpretations. In this context, we separately analyzed the fruit juice consumption (natural orange and other natural fruit juices). The CRP and homocysteine concentrations as well as mRNA expression of *IL1R1*, *IL6*, and *TNFα *genes were statistically significant (*P *for trend < 0.05) lower in those subjects included in the third tertile of energy-adjusted fruit juice consumption (> 100 ml/d), adjusting for gender, age, BMI, energy intake, smoking habit, physical activity counts, NEFA concentration, and systolic blood pressure.

In addition, the separate regression analyses of the influence of the smoking status (never, former, and current smokers) or including the smoking habit categorized by number of cigarettes/day (1) 0-5 or ≥ 5 cigarettes/d; 2) 0-10 or ≥ 10 cigarettes/d) as covariates, produced the same outcome concerning the inflammatory markers. Finally, when we performed linear regression analysis, including the waist circumference as independent variable, the results followed the same trends and remained statistically significant (data not shown).

## Discussion

A high intake of fruits and vegetables has been inversely associated with metabolic syndrome features [5,6], cardiovascular diseases [31,32], and total mortality [33], while greater values of proinflammatory markers such acute-phase proteins, cytokines, and adhesion molecules have been directly associated with chronic disorders [2,3]. In this context, the potential relationships between specific dietary factors and proinflammatory markers are being currently investigated [19-21,34]. In this context, nutrigenomic approaches have been performed as a potential useful tool to increase fundamental knowledge concerning the interactions between diet and gene expression [13,14]. Thus, this study found, apparently for first time, that healthy adults with a high consumption of fruits and vegetables had lower *ICAM1, IL1R1, IL6, TNFα*, and *NFκB1(p50) *gene expression in PBMC.

An altered expression of *ILs*, *TNFα*, *ICAM1 *genes and of their respective receptors in adipose tissue as well as in PBMC has been implicated in the higher risk of suffering metabolic syndrome and cardiovascular disease [3,4,35]. In addition, NFκB is a redox-sensitive transcription factor implicated in the transmission of different signals from the cytoplasm to the nucleus, which are involved in the regulation of inflammatory and immune genes, apoptosis, and cell proliferation [36]. In this regard, the activation of this transcription factor has been involved in atherosclerosis [37]. Therefore, the reported inverse association between fruits and vegetable consumption and the selected proinflammatory gene expression measurements suggest a new clinically relevant mechanism concerning the prevention of subclinical inflammation status in healthy adults by a high intake of fruits and vegetables.

In this study, we also found a statistically significant inverse association of fruit and vegetable consumption with CRP and homocysteine concentrations. These results are consistent with some earlier cross-sectional studies carried out on children and adolescents [38,39] as well as middle-age adults [7,40]. Moreover, in a randomized crossover study, the addition of vegetables to the diet has been able to reverse the increase in circulating vascular adhesion molecule-1 (VCAM1), ICAM1, IL6, and TNFα levels, as induced by a single high-fat (saturated) meal consumption [11]. Furthermore, in a randomized controlled 4-week trial, a high consumption (eight vs. two servings/day) of fruits and vegetables also significantly reduced CRP levels [12].

The anti-inflammatory mechanisms related to fruit and vegetable consumption are still unclear. Fruits and vegetables are sources of essential nutrients, which could be implicated in inflammation and oxidative stress reductions [10,41]. For example, the intake of folate, vitamin C, and magnesium, for those fruits and vegetables have relevant content, have been associated with lower homocysteine, CRP, IL6 and E-selectin concentrations [42-44]. Fruits and vegetables also are important sources of dietary fiber, which appears to have an anti-inflammatory role [10,41]. Also, dietary fiber intake could participate in weight control and favor weight loss, hypoglycemic actions and hypolipidemic effects [45]. In addition, butyrate production after the consumption of dietary fiber could have an inhibitory effect on the NFκB and a stimulatory effect on PPAR-α activation, with subsequent lower expression of *ICAM1 *and *VCAM1 *genes [46], although this mechanism deserves further research. Furthermore, fruits and vegetables contain several flavonoids and carotenoids with recognized antioxidant properties, which may play a role in the inverse relationship between intake of fruits and vegetables and inflammatory status [9,38,47]. In fact, several *in vitro *studies have evidenced an anti-inflammatory effect of flavonoids and carotenoids, by an inhibition of NFκB activity, through suppressing the activation-related phosphorylation, and inhibiting the nuclear translocation [48-50].

In this study, we also found inverse associations of dietary fiber and TAC with plasma concentrations and the gene expression of certain proinflammatory markers, suggesting a participation of these specific dietary factors in the beneficial effect of fruit and vegetable consumption. The gene expression of investigated proinflammatory markers had statistically significant lower values across tertiles of fruit and vegetable consumption, independently of dietary fiber and dietary TAC. In addition, the inverse associations of fruit and vegetable consumption with proinflammatory gene expression maintained the trend and the statistical significance after including others specific dietary factors such as plant protein, potassium, magnesium, total fat, polyunsaturated fatty acid and saturated fatty acid. In this context, our findings suggest that additive and synergistic effects of bioactive compounds provided by fruits and vegetables as responsible for the antioxidant and anti-inflammatory activities of these foods.

Interestingly, we found lower values to BMI, waist circumference, and blood pressure in those participants who were in the highest tertile of fruit and vegetable consumption. These results are in accordance with findings obtained in the SUN Study [5,51] and in The Dietary Approaches to Stop Hypertension (DASH) trial [52,53]. In turn, it has been also reported a positive association of proinflammatory marker concentrations and gene expression in PBMC with body fat distribution [54] in healthy young adults. Thus, the relationships between fruit/vegetable consumption and proinflammatory markers could be biased by body fat composition. When we controlled our analyses by these potential confounding factors, including BMI or waist circumference as continuous independent variables, the hypothesized associations between fruit and vegetable consumption and proinflammatory markers maintained the trend and the statistical significance, suggesting that the effects of fruit and vegetable consumption that we found in this study were not totally explained by differences in body fat variables. Our study had certain limitations. First, since the nature of this study is cross-sectional, we cannot prove that the reported associations are causal because residual confounding may have affected the observed associations. However, we controlled for the more important known factors that affect proinflammatory gene expression. Second, dietary exposures can be misclassified despite the good correlation between food frequency questionnaires and usual diet [55] but the dietary questionnaire is validated [16], and has been successfully applied to investigate the relationship between dietary factors and inflammatory markers [19-21]. Third, a higher consumption of oranges and tomatoes as well as the contribution of the remaining food items to the fruit and vegetable consumption could be influenced by the season in which the questionnaires were completed [56], although the SUN food-frequency questionnaire has presented a good reproducibility [55] in different circumstances. Fourth, although the sample size is adequate from the standpoint of initial association discovery, further replication in independent and larger samples will be convenient for a future translational application at a population level.

## Conclusion

In this translational study, the fruit and vegetable consumption was inversely associated with mRNA expression of certain proinflammatory markers from PBMC in healthy young adults, suggesting a beneficial effect of high fruit and vegetable consumption on decreasing proinflammatory status and providing new light for the nutrigenomic involved-mechanisms as well as new tools for the assessment of nutrient-gene interactions.

## Abbreviations

BMI: Body mass index; CRP: C-reactive protein; DASH: The Dietary Approaches to Stop Hypertension; ICAM1: intercellular adhesion molecule-1; IL1R1: interleukin-1 receptor-1; IL: interleukin; MET: activity metabolic equivalent; mRNA: messenger RNA; NEFA: non-esterified fatty acid; NFκB1: nuclear factor-nuclear-kappa-B (p50); PBMC: peripheral blood mononuclear cells; RELA: nuclear factor-nuclear-kappa-B (p65); SUN: Seguimiento Universidad de Navarra; TAC: total antioxidant capacity; TNFα: tumor necrosis factor-alpha.

## Competing interests

The authors declare that they have no competing interests.

## Authors' contributions

HHMH: Design, field work, data collection, analysis, and writing of the manuscript. BP: Design, field work, and data collection. MZ: project co-leader, design, financial management, and editing the manuscript. JAM: project leader, general coordination, design, financial management, and editing of the manuscript. All authors read and approved the final manuscript.
